# Glucose-6-Phosphate Dehydrogenase: Update and Analysis of New Mutations around the World

**DOI:** 10.3390/ijms17122069

**Published:** 2016-12-09

**Authors:** Saúl Gómez-Manzo, Jaime Marcial-Quino, America Vanoye-Carlo, Hugo Serrano-Posada, Daniel Ortega-Cuellar, Abigail González-Valdez, Rosa Angélica Castillo-Rodríguez, Beatriz Hernández-Ochoa, Edgar Sierra-Palacios, Eduardo Rodríguez-Bustamante, Roberto Arreguin-Espinosa

**Affiliations:** 1 Laboratorio de Bioquímica Genética, Instituto Nacional de Pediatría, Secretaría de Salud 04530, Mexico; 2 Consejo Nacional de Ciencia y Tecnología (CONACYT), Instituto Nacional de Pediatría, Secretaría de Salud 04530, Mexico; racastilloro@conacyt.mx; 3 Laboratorio de Neurociencias, Instituto Nacional de Pediatría, Secretaría de Salud 04530, Mexico; america_vc@yahoo.com.mx; 4 Consejo Nacional de Ciencia y Tecnología (CONACYT), Laboratorio de Bioingeniería, Universidad de Colima, Colima 28400, Mexico; hjserranopo@conacyt.mx; 5 Laboratorio de Nutrición Experimental, Instituto Nacional de Pediatría, Secretaría de Salud 04530, Mexico; dortegadan@gmail.com; 6 Departamento de Biología Molecular y Biotecnología, Instituto de Investigaciones Biomédicas, Universidad Nacional Autónoma de México, Mexico City 04510, Mexico; abigaila@correo.biomedicas.unam.mx; 7 Laboratorio de Inmunoquímica, Hospital Infantil de México Federico Gómez, Mexico City 06720, Mexico; beatrizhb_16@comunidad.unam.mx; 8 Colegio de Ciencias y Humanidades, Plantel Casa Libertad, Universidad Autónoma de la Ciudad de México, Mexico City 09620, Mexico; edgar.sierra@uacm.edu.mx; 9 Departamento de Química de Biomacromoléculas, Instituto de Química, Universidad Nacional Autónoma de México, Circuito Exterior s/n, Ciudad Universitaria, Mexico City 04510, Mexico; e-rodriguez-bustamante@ciencias.unam.mx (E.R.-B.); arrespin@unam.mx (R.A.-E.)

**Keywords:** glucose-6-phosphate dehydrogenase (G6PD) enzyme, mutations, bioinformatics tools, three-dimensional structure, clinical manifestations

## Abstract

Glucose-6-phosphate dehydrogenase (G6PD) is a key regulatory enzyme in the pentose phosphate pathway which produces nicotinamide adenine dinucleotide phosphate (NADPH) to maintain an adequate reducing environment in the cells and is especially important in red blood cells (RBC). Given its central role in the regulation of redox state, it is understandable that mutations in the gene encoding G6PD can cause deficiency of the protein activity leading to clinical manifestations such as neonatal jaundice and acute hemolytic anemia. Recently, an extensive review has been published about variants in the *g6pd* gene; recognizing 186 mutations. In this work, we review the state of the art in G6PD deficiency, describing 217 mutations in the *g6pd* gene; we also compile information about 31 new mutations, 16 that were not recognized and 15 more that have recently been reported. In order to get a better picture of the effects of new described mutations in *g6pd* gene, we locate the point mutations in the solved three-dimensional structure of the human G6PD protein. We found that class I mutations have the most deleterious effects on the structure and stability of the protein.

## 1. Gene Structure of Glucose-6-Phosphate Dehydrogenase (G6PD)

**Figure 1:**
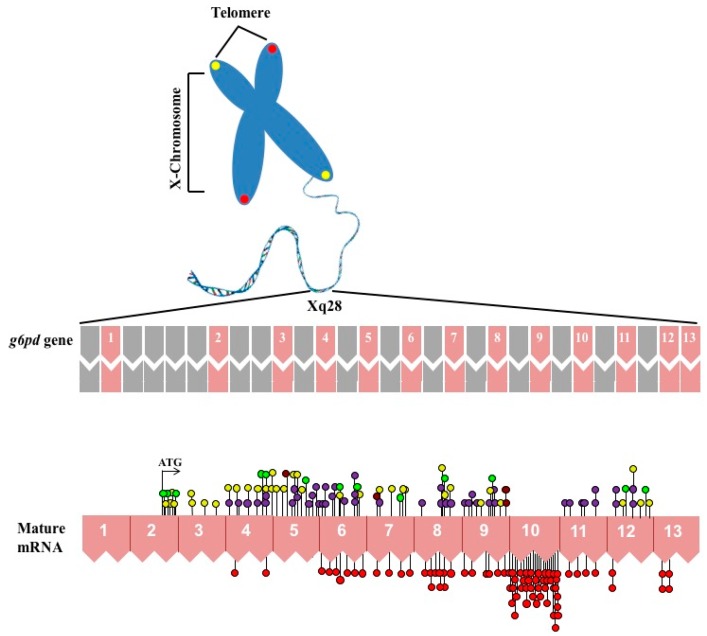
Schematic overview of X chromosome and distribution of mutations in *g6pd* gene coding sequence. The **top** shown the introns and exons that are shown in **gray** and **pink** color boxes, respectively. The numbers (1–13) indicate exons of the human *g6pd* gene. In the **bottom**, the mRNA is schematized and all the single nucleotide substitutions (missense variants) are showed. The **red** circles are mutations associated with chronic nonspherocytic hemolytic anemia. **Purple** circles showed the Class II mutations. Class III mutations are shown in **yellow** circles. Class IV mutations are shown in **brown** circles and the unnamed reported class mutations are shown in bright **green** circles.

The G6PD (EC 1.1.1.49) is an X-linked cytosolic enzyme that is present in all forms of life from prokaryotes to animals [1,2,3]. In humans, the gene encoding G6PD is located near the telomeric region of the distal arm of the X chromosome which is well known as a hot spot [1,3,4,5] (band Xq28) (Figure 1) and belongs to a group of genes that includes fragile X [6], color vision [7,8], hemophilia A [9], and congenital dyskeratosis [10]. The complete sequence of the *g6pd* gene has a size of 18.5 Kb and consists of 13 exons and 12 introns encoding a product of 1545 bp [11]. In an unusual way, the first 600 bp of the 5′ of the *g6pd* gene, which correspond to exon 1 and part of exon 2, are not translated and as a consequence the start codon ATG is located in the base 115 of the 127 bp of exon 2 (Figure 1). This untranslated 5′ sequence contains two ATG codons out of reading frame and exhibits two sub-regions with a high level (80%) of guanine and cytosine which is a common characteristic among genes with constitutive expression [3,4]. Regarding the promoter region of the *g6pd* gene, several features have been identified: (i) high GC content (over 70%); (ii) the absence of CAAT element often located in the position −70 to −90 of various eukaryotic genes (essential site for the transcription process); (iii) the substitution of classical TATA box by ATTAAAT sequence located at −202 bp from the ATG start codon; and (iv) the presence of at least nine CCGCCC sites, which appear to be involved in gene regulation [3,4]. The *g6pd* gene product is a 59 KDa protein with 514 amino acids [12]. Mutations that are distributed throughout the *g6pd* gene lead to a hereditary disease at the population level known as G6PD deficiency (Figure 1).

## 2. Epidemiology G6PD Deficiency

The deficiency of G6PD has been recognized as the most common enzymopathy, affecting near 400 million people worldwide [13]. G6PD deficiency is more commonly expressed in males compared to females [14,15,16] and occurs most frequently in Africa, Asia, the Mediterranean, and the Middle East. In the United States, this deficiency principally affects men of African and Mediterranean ethnic origins, with a prevalence of approximately 10% [17,18]. Interestingly, the prevalence of G6PD deficiency correlates with the geographical distribution of malaria, leading to postulate that G6PD deficiency gives a partial protection against this infection [18,19].

G6PD variants are generally classified according to the severity of the G6PD deficiency that accompanies the enzyme activity and hematological parameter of the patients, ranging from the most severe manifestations with less than 5% residual activity (Class I) to the mildest form (Class V) [20,21]. The prevalence of this deficiency is variable, with frequencies from 2% to 20% in Greece, Turkey, and Italy; but increased as much as 70% in groups of Kurdish Jews [18]. Recently, a Bayesian geostatistical model estimated the prevalence of G6PD deficiency worldwide [22]. This map presented the allelic frequency of the phenotypic deficiency, which is the prevalence of the disease in men. The results of this study showed that in Latin America (LA), the prevalence of G6PD deficiency is lower compared with other regions such as sub-Saharan Africa or Asia [22,23]. Interestingly, G6PD deficiency in LA varies significantly from region to region even in the same country. These variations may be due to the genetic heterogeneity between different populations, the disparity of the diagnostic methods used, or the inequality of epidemiological studies in many areas of LA [22,23].

## 3. Biochemical Aspects of the G6PD (Metabolic Function and Deficiency)

**Figure 2:**
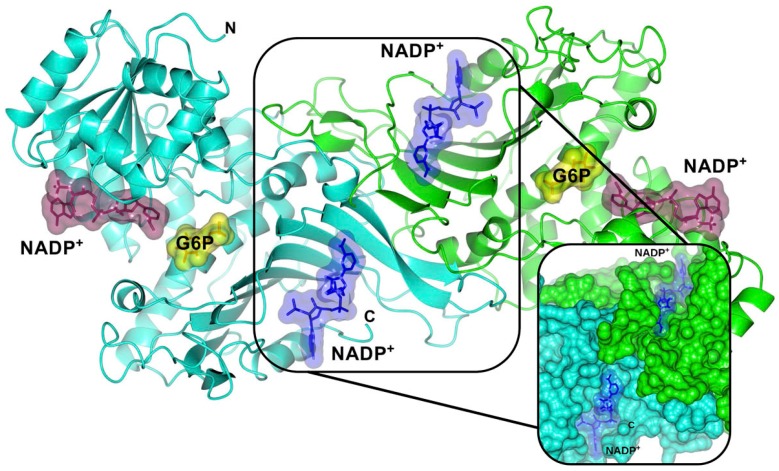
Crystallographic structure of the human wild-type (WT) G6PD enzyme (PDB entries 2BHL and 2BH9), showing the structural NADP^+^ (**blue** molecular surface), catalytic NADP^+^ (dark **purple** molecular surface), and G6P substrate (**yellow** molecular surface) in the dimer. The two monomers are shown in **cyan** and **green**. Right inset, close-up of the dimer interface and both structural NADP^+^ molecules. The figure was prepared using Collaborative Computational Project Number 4-Molecular Graphics (CCP4mg) (Didcot, UK) [28]. The same color code for G6PD enzyme is used in all other figures.

G6PD enzyme is active in red blood cells (RBCs) as tetramer or dimeric form. Dimer structures of the two subunits in the enzyme are symmetrically located across a complex interface of β-sheets and each subunit binds to a NADP^+^ molecule for its structural stability [24,25] (Figure 2). This enzyme is the rate-limiting enzyme of the pentose phosphate pathway (PPP) that breaks down glucose, promotes the oxidation of β-d-glucose-6-phosphate to d-glucono-1,5-lactone-6-phosphate, and produces a reduced form of nicotinamide adenine dinucleotide phosphate (NADPH) as a byproduct in oxidative phase [26]. d-glucono-1,5-lactone-6-phosphate is hydrolyzed to generate 6-phosphogluconate and then decarboxylated by 6-phosphogluconate dehydrogenase (6PGD) enzyme to yield the five-carbon molecular ribulose 5-phosphate (Ru5P)—a precursor of DNA, RNA, and ATP—with the concomitant generation of another NADPH molecule [3,27].

**Figure 3:**
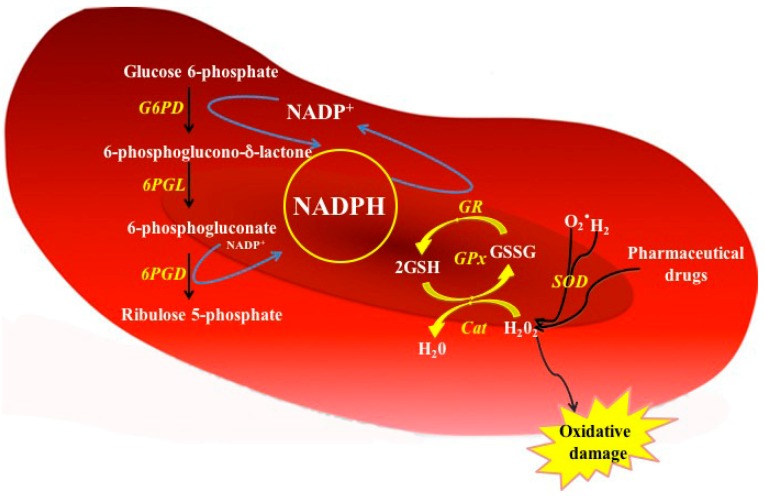
Function of G6PD enzyme in the PPP from red blood cells. In G6PD-normal red cells, the NADPH is produced by the action of glucose 6-phosphate dehydrogenase (G6PD) and 6-phosphogluconate dehydrogenase (6PGD) enzymes. The NADPH serves as proton donor to regenerates the GSSG oxidized. Cat = Catalase; GPx = Glutathione peroxidase; GR = Glutathione reductase; G6PD = glucose 6-phosphate dehydrogenase; 6PGL = 6-phosphogluconolactonase; 6GPD = 6-phosphogluconate dehydrogenase; SOD = Superoxide dismutase; GSH = Reduced glutathione; GSSG = Oxidized glutathione; H_2_O_2_ = Peroxide; O_2_^−^ = Superoxide.

It is noteworthy that, unlike other cells types, RBCs do not contain mitochondria and therefore the PPP pathway is the only source of NADPH, which plays a key role in the protecting cells against oxidative damage due to reactive oxygen species (ROS) [29]. Because the important function of NADPH is scavenging cellular ROS, NADPH is involved in at least three antioxidant pathways: the glutathione, thioredoxin, and glutaredoxin cycles (Figure 3). In the first pathway, the electron of NADPH passes to glutathione dimers (GSSG) during the reaction catalyzed by glutathione reductase enzyme that produces two reduced glutathione monomers (GSH) providing the first line of defense against ROS [30]. Moreover, glutathione peroxidase (GPX) removes peroxide from RBCs using GSH as substrate, while the NADPH is required to reduce GSSG oxidized and the sulfhydryl groups of some necessary proteins for the protection against oxidative stress. The RBCs that cannot eliminate this stress suffer hemolysis [3,14,19,31].

## 4. Molecular Characterization of G6PD Variants

More than 400 variants of G6PD have been described based on their biochemical and physicochemical characteristics [2,32,33]. Minucci et al. [34] reported, up to date, around 186 mutations at DNA level indicating that this disease is heterogeneous: among these, 159 (85.4%) are single nucleotide substitutions (missense variants), 15 (8.0%) are multiple mutations (two or more substitutions), 10 (5.3%) are deletions, and two (1.0%) are mutations affecting introns [34,35,36]. In this review, we add 31 new mutants of G6PD enzyme (Table 1). Based on mutation type, 23 have a variation in single nucleotide substitutions (missense variants), one has a deletion, four belong to multiple mutations, and three are novel mutants that affect the introns of gene. Taking account these new variants included in this review, the number of reported G6PD mutations is 217, being as follows: 182 (83.9%) are single nucleotide substitutions (missense variants), 19 (8.7%) are multiple mutations (two or more substitutions), 11 (5.1%) are deletions, and five (2.3%) mutations affect the introns. Interestingly, we found 16 mutations corresponding to single nucleotide substitutions (missense variants) that were previously reported before in Minucchi’s review [34] and that were not considered as the G6PD Class I mutants: Zacatecas (Arg257Leu; exon 7) [37], Palermo (Arg257Met, exon 7) [38], Hamburg (Pro276Leu, exon 8) [39], Veracruz (Arg365His, exon 10) [37], Yucatan (Lys429Glu, exon 10) [37], Tennessee (Leu422Val, exon 10) [40], and one deletion named G6PD Taif (174Gly, exon 6) [41] (Table 1).

**Figure 4:**
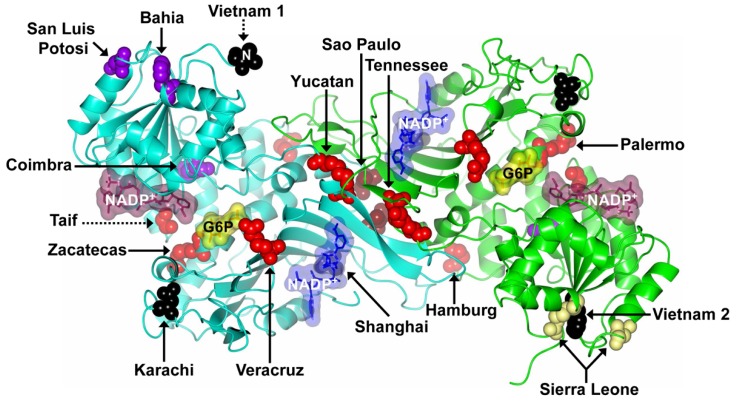
Mutations in the G6PD protein that have not been considered in the most recent review [34]. Structure of human WT G6PD enzyme (PDB entries 2BHL and 2BH9) indicating the location of Class I–IV mutations (missense variants) in the dimer (solid arrows). The Class I, II, III, and IV mutations are shown as **red**, **purple**, **yellow**, and **brown** spheres, respectively. The unnamed reported class mutations are shown as **black** spheres. Note that although the mutants are located on equivalent positions of G6PD dimer, Zacatecas, Palermo, Bahia, Vietnam 1 and 2, Sierra Leone, and San Luis Potosí mutants are shown in only one of the monomers. Also note that the Taif mutant is a deletion (dotted arrow) and Vietnam 1 mutant is represented by Val27 residue, since no electron density was observed for the 26 N-terminal residues where the Vietnam 1 mutant is located (dotted arrow). The figure was prepared using CCP4mg [28].

The Class I G6PD Zacatecas mutant (Arg257Leu) was detected in a 12-year-old boy from the Mexican state of Zacatecas with neonatal jaundice and hemolytic crisis. This variant has a substitution of guanine for thymine (G > T) at nucleotide (nt) 770 (exon 7) leading to the replacement of arginine by leucine 257 (R → L) that, according to tridimensional structure is located at a distance of ~9 Å from the substrate-binding site of the β-d-glucose-6-phosphate (G6P) [37,42] (Figure 4). It is important to mention that this mutation is located in the same codon as the G6PD Wayne (R257G) that was previously characterized as a Class I mutant [43]. However, Monteiro’s review [44] classified at the G6PD Zacatecas mutation as a Class II mutant. G6PD Veracruz mutation was detected in anonymous blood samples from blood donors and has a substitution of guanine for adenine G1094A that changes the amino acid Arg 365 to His. The G6PD Yucatan mutation presents a shift of adenine for guanine (nt 1285A > G, K429E) and is located in exon 10. All these mutants were identified in a project performed to determine the molecular basis of G6PD deficiency in Mexico, including nearly 5000 individuals from the general population and patients with hemolytic anemia belonging to at least 14 States from Mexico [37].

G6PD Palermo mutation presents a transversion of the nucleotides C769A and G770T in exon 7 and replaces arginine for methionine in codon 257, which may lead to changes in the protein structure causing chronic hemolytic anemia [38] (Figure 4). Kordes et al. [39] reported the Class I G6PD Hamburg mutant involving a substitution of cytocine for thymine (C > T) at nucleotide 827 (exon 8) (Pro276Leu) and was detected in a Caucasian neonate with chronic nonspherocytic hemolytic anemia (Figure 4). Another variant is the Class I G6PD Tennessee identified by McDade et al. [40] in an African-American male, it consists of a new mutation in Exon 10, C1465G, producing a change in leucine to valine at codon 422 (Figure 4). Finally, G6PD Taif mutation has three base deletions at position 516–518 in exon 6 resulting in the loss of the amino acid Gly174 [41]. This mutation is unique because it is the only known deletion identified in this region that causes chronic hemolytic anemia (Figure 4).

Besides the mutants described by Minucchi’s review [34], we include the following new variants: Class II G6PD Bahia (Phe66Thr, exon 4) [43], San Luis Potosi (Asn126Tyr; exon 5) [37], Coimbra (Arg198His, exon 6) [45], a class III variant with double mutation; G6PD Sierra Leone (Arg104His, Asn126Asp, exon 5) [46], a class IV G6PD San Paulo mutant (Ile220Met, exon 7) [47]. Finally, we found four mutants that did not classified according to their residual enzyme activity: G6PD Shanghai (Ala231Pro, exon 7) [48], G6PD Karachi (Asp325Asn, exon 9) [49], G6PD Vietnam 1 (Glu3Lys, exon 2) [50], and G6PD Vietnam 2 (Phe66Cys, exon 4) [50] which are shown in the Figure 4.

The G6PD San Luis Potosi has a punctual mutation of adenine to thymine A376T that generates the substitution of 126 Asn to Tyr (Figure 4) [37] and was detected in an anonymous blood samples (Figure 4). Blood sample from the subject with this variant showed a decreased fluorescence in the screening test for G6PD deficiency and 47% of residual red cell enzyme activity versus those observed in their correspondent control blood samples from non-deficient subjects [37]. Whereas, Chalvam et al. [45] reported that G6PD Coimbra mutation detected among the tribal groups of the Nilgiris in Southern India, suffers a substitution of guanine for adenine (593G > A) on exon 6 switching Arg 198 by His. G6PD Coimbra is very close to the G6PD Mediterranean in exon 6 and has similar kinetic properties [45]. Other mutation, the Class II G6PD Bahia mutant was reported by Pereira et al. [51], this mutant has a substitution of thymine for adenine (T > A) at nucleotide 197 (exon 4) (Phe66Thr) and was found in five neonates of Salvador in the Northeastern Brazilian estate Bahia (Figure 4).

Jalloh et al. [46] reported the Class III G6PD Sierra Leona variant, which has double mutation in exon 5 (311G > A and 376A > G) leading to the change of amino acid 104 Arg → His and 126 Asn → Asp, respectively. This mutation drastically reduces G6PD activity [46]. In addition, Oliveira et al. [47] reported a novel Class IV G6PD Sao Paulo variant 660C > G that was detected in exon 7, this mutation led to replacement of isoleucine by methionine (I220M) whose location is near the dimer interface (Figure 4). It is interesting to note that this mutation was detected among adults.

**Figure 5:**
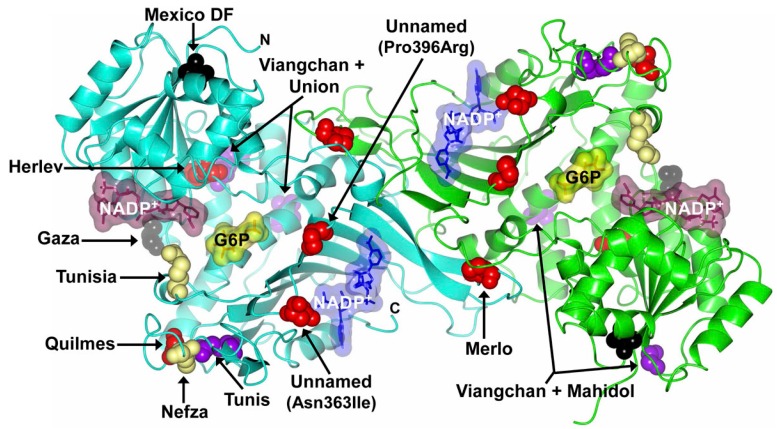
Recent described mutations in the G6PD protein. Structure of human WT G6PD enzyme (PDB entries 2BHL and 2BH9) indicating the location of Class I–IV mutations (missense variants) in the dimer. The Class I, II, III, and IV mutations are shown as **red**, **purple**, **yellow**, and **brown** spheres, respectively. The unnamed reported class mutations are shown as **black** spheres. Note that although most of the mutants are located on equivalent positions of G6PD dimer, Viangchan + Mahidol and Viangchan + Union mutants are shown in only one of the monomers. The figure was prepared using CCP4mg [28].

Additionally, the four mutants that were not described by Minucchi’s review [34] and that have not been classified according to their residual enzyme activity are the mutants G6PD Vietnam 1 (G7A; Glu3Lys) and Vietnam 2 (T197G; Phe66Cys) detected in the Vietnamese population with hemoglobinuria [50] (Figure 4). The G6PD Shanghai is a novel missense mutation (G691C) in exon 7 of the G6PD producing an Ala231Pro substitution and leading to significantly decreased of G6PD activity in red blood cells. Finally, the G6PD Karachi mutant was detected in the Pakistani population with a change of guanine by adenine G > A in the nucleotide 973 exon 9, with a predicted amino acid change of Asp325Asn (Asp325Asn) [49].In addition, we also include the most recent described mutations for *g6pd* gene (Table 1). Into the Class I G6PD mutants are the Quilmes (Ser332Phe, exon 9) [52], Merlo (Pro409Gln, exon 10) [52], Herlev [53], and two unnamed mutants (Pro396Arg, exon 10) [54] and (Asn363Ile, exon 10) [55]. Also we incorporate one mutant Class II named G6PD Tunis (Gln307Pro, exon 9) [54] and a Class III G6PD Nefza (Leu323Pro, exon 9) [56] (Figure 5). Furthermore, two double mutants Class II/III G6PD Viangchan + Mahidol (Val291Met, Gly163Ser, exon 9 and 6) and G6PD Viangchan + Union (Val291Met, Arg454Cys, exon 9 and 11) were reported by Nantakomol et al. [57] (Figure 5). In addition, two novel mutants: Mexico DF (Thr65Ala, exon 2) [58] and Gaza (Ser179Asn, exon 6) [59] that were not classified according to their residual enzyme activity are considered (Figure 5). Finally, we included a novel mutant that affects the introns named G6PD Qingzhen (IVS5-1G > A) reported by Wei-Liang et al. [60].

More recently, the Class I G6PD Merlo and Quilmes mutants were found in Argentine pediatric boys with chronic nonspherocytic hemolytic anemia. G6PD Quilmes Variant 995C > A was found in exon 9 and determines the Ser332Phe amino acid change. Variant Merlo 1226C > A, is located in exon 10 and is associated with the Pro409Gln amino acid change [56] (Figure 5). The unnamed mutant (Pro396Arg, exon 10) was identified in a 20-month-old boy with hemolytic anemia whose enzymatic activity was severely decreased [54]. In addition, Warny et al. [53] described G6PD Herlev variant 592C > A (Arg198Ser) associated with severe enzyme deficiency, prolonged neonatal hyperbilirubinemia, and nonspherocytic hemolytic anemia in a Danish descent male infant who on the second day of life developed jaundice peaking at 67 h that decreased upon application of double-sided phototherapy. Recently, a novel unnamed mutation (1088A > T) was identified in a male infant patient aged 16 month-old that predicted an Asn → Ile substitution at codon 363. The variant caused by this mutation had reduced enzymatic activity, belonging to WHO Class I [55].

Another two double mutants in Thai population were described; 6PDViangchan + Mahidol and G6PD Viangchan + Union [57] (Figure 5). These double mutants should be classified as a severe G6PD deficiency. However, clinical data is also required to classify them in a Class I or Class II G6PD deficiency. Later, two new variants were characterized on exon 9 and were found during the screening of a large cohort of G6PD deficient patients that was performed in Tunisia. The first one was named G6PD Nefza and carries the c.968T > C: p.323 Leu → Pro mutation (Figure 5), this variant was found in an 18 year old male referred to the laboratory for hemolytic anemia. The second mutation, named G6PD Tunis carries the c.920A > C; p.307Gln → Pro mutation, and was found in a 54 year old female presenting hemolytic anemia and paleness [56].

Furthermore, Garcia-Magallanes et al. [58] reported the presence of a novel mutant G6PD Mexico DF that was identified in a Mexican individual from the northern Mexico; which presents the substitution of 193A > G that changes the amino acid threonine for alanine at position 65 (exon 4) (Figure 5). The Thr65Ala mutation might lead to an unstable coenzyme union site region of the enzyme, due to the combined effect of the suppression of a peptide backbone H-bond and by disrupting the domain packing. Furthermore, Sirdah et al. [59] reported the G6PD Gaza mutant (unclassified according to their residual enzyme activity) that was found in a Palestinian girl (38 months old) as a heterozygous genotype, presenting acute hemolytic anemia and G6PD deficiency. This variant is characterized by a G > A transition mutation at nucleotide 536, which changes serine 179 to asparagine. Besides, the G6PD Qingzhen which is a mutation affecting the introns, was detected in a 2.5 year old male patient of the Yi ethnic group in the Qingzhen city, Guizhou province, in China. The patient presented acute jaundice, anemia, wine urine, emesis, low-grade fever, and the occasional headache. Mutation analysis of G6PD revealed that the patient had a novel subtle splice-site mutation (IVS5-1G > A) [60] (Figure 5). In addition, two mutations affecting introns were reported by Bendaou et al. [61], where the first was the G6PD mutation IVS-V 655C > T, found in four female subjects with mild deficiency of class III variant. The second mutation affecting the introns was the IVS-VIII 43G > A, found in three male subjects with a mild deficiency of the class III variant.

It is interesting to note that all known mutations have been found to affect the coding regions of the gene and none described in the regulatory regions [62]. Because mutations can alter the cellular process that generally trigger a metabolic disease, we located the point mutations in the structure of human WT G6PD enzyme and analyzed the impact of several mutations reported in this review. For this purpose, we used the native crystals structure of G6PD as a template. The model predicts the substitutions of all analyzed amino acids affecting the coding regions of the gene. However, each mutation may produce clinical phenotypes such as hemolytic anemia or chronic non-spherocytic hemolytic anemia. This data suggests that the change in protein function is not straightforward.

**Figure 6:**
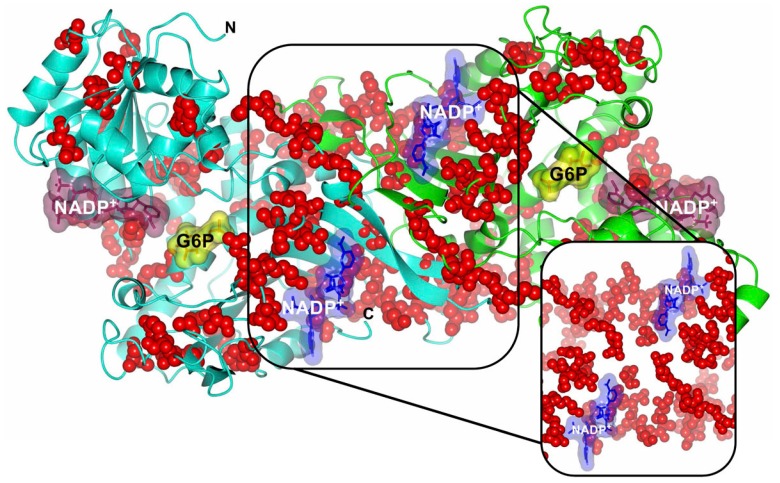
Structure of human WT G6PD enzyme (PDB entries 2BHL and 2BH9) indicating the location of Class I mutations in the dimer. Note that all mutations are located on equivalent positions of G6PD dimer. Right inset, close-up of Class I (**red** spheres) mutations located in both the dimer interface and near the structural NADP^+^ molecules. The figure was prepared using CCP4mg [28].

The analysis of Class I G6PD mutants using the X-ray structure (Figure 4 and Figure 5) showed that G6PD Zacatecas, Palermo, and Taif are located near the active site; while that other (Class I) mutations as Veracruz, Tennessee, San Paulo, Shanghai, Unnamed (pro396Arg), and Unnamed (Asn363Ile) are located near to the dimerization interface in proximity with structural NADP^+^ binding. Mutant Class I as Hamburg, Yucatan, Quilmes, and Merlos are located in different parts of the tridimensional structure. Although these mutations are distributed throughout the all-coding region of the *g6pd* gene, there is a group of mutations that are associated with Class I deficiency having severe clinical manifestations as hemolytic anemia and chronic nonspherocytic hemolytic anemia (CNSHA) with a severe enzyme deficiency. Class I mutations more frequently affect the exons 6, 8, 10, and 13 encoding the regions that bind the enzyme substrate, dimer interface, and NADP^+^ structural site, respectively [34,63,64,65] (Figure 6).

## 5. Clinical G6PD Deficiency

The clinical manifestation of G6PD deficiency in humans has a broad clinical spectrum ranging from almost asymptomatic individuals to those with severe neonatal jaundice, acute hemolytic episodes, and chronic non-spherocytic hemolytic anemia suggesting that gene-environment interactions may influence the clinical outcome of G6PD deficiency [66,67,68]. The severity of the clinical manifestation often correlates with the grade of the enzymatic dysfunction. A large majority of G6PD deficiencies are asymptomatic most of the time, until they are exposed to a hemolytic trigger. Accordingly to clinical manifestations the G6PD deficiency can be divided in three groups: neonatal jaundice, hemolytic anemia (drug-induced hemolysis, diabetes mellitus-induced hemolysis, and infection-induced hemolysis), and chronic nonspherocytic anemia.

### 5.1. Neonatal Jaundice

The most severe clinical symptom of G6PD deficiency is neonatal jaundice (NNJ), which peaks two to three days after birth [69]. In newborns with Class I G6PD deficient variants, the jaundice could be considered dangerous and cause kernicterus [70] and permanent neurological damage [71]; which in many cases provokes the death of the patient [72]. However, not all neonates with NNJ are G6PD deficient, because many of the mechanisms involved in the depuration of bilirubin are not fully developed at birth. However, in infants with G6PD deficiency, the prevalence of neonatal jaundice is two-fold higher than in the general population [17] and more frequent and severe in premature infants [23].

### 5.2. Hemolytic Anemia

The acute hemolytic anemia (AHA) is the most common manifestation of the deficiency, which is originated when the RBCs are under oxidative stress and may be triggered by a range of exogenous agents as fava beans, drugs, or infections, causing intravascular hemolysis and jaundice [73]. The most severe outcome of AHA is acute renal failure [30]. Furthermore, unrelated events to these agents have been reported such diabetes, myocardial infarction, or even vigorous exercise which can trigger hemolysis [70,74,75]. Mutants with these clinical manifestations are the G6PD Tunis and Sierra Leone variants that were found in a 54 year-old female and 15-year-old boy presenting hemolytic anemia and a very low residual enzyme, respectively [46,56].

The relationship between the ingestion of dry grains or frozen fava bean (*Vicia faba*) and its pathological effects (Favism) had been observed for centuries in the Mediterranean countries [69]. A example of this type of clinical manifestation is the mutant G6PD Nefza that was found in an 18-year-old male with hemolytic anemia triggered by fava beans. Furthermore, the patient reported a neonatal jaundice in infancy. Biochemical data revealed a decreased enzyme activity (51% of residual activity, in period out of hemolysis) [56]. Favism is most common seen in children between the ages of two to five, and is also two to three times more common in boys than in girls [69]. Since then, some other drugs (antimalarial, sulfonamides, sulfones, nitrofurantoin, etc.) have been linked to the development of acute hemolysis in G6PD deficient patients [30]. Besides, infection as hepatitis viruses A and B, cytomegalovirus, pneumonia, and typhoid fever have been previously attributed as another important trigger of AHA [30]. However, the exact mechanism by which the infections encourage the hemolysis is unknown, probably is due to the production of oxidizing species by leukocytes that could cause oxidative stress in the erythrocytes [76]. Finally, it has been proposed that the damage in erythrocytes produced for oxidative damage of drugs is similar to favism, because the fava beans contain oxidizing compounds as divicine, isouramil, and convicine.

### 5.3. Chronic Nonspherocytic Hemolytic Anemia (CNSHA)

In some patients, the presence of the most severely affected variants (Class I) as G6PD Zacatecas [37], Hamburg [39], Quilmes [52], Veracruz [37], Merlo [52], Yucatan [37], Tennessee [42], unnamed A1088T [55], and unnamed C1187G [54] mutants presented acute hemolytic anemia and jaundice. Furthermore, the G6PD Taif mutant is unique due that it is the only known deletion in this region closed to the putative G6P-binding domain. While the novel splice G6PD Qingzhen mutation (IVS5-1G > A) was found in a 2.5 year-old male patient. Although this mutant was not classified according to their residual enzyme activity, the patient showed acute jaundice, anemia, wine urine, emesis, low-grade fever, occasional headache, and bellyache after eating broad beans [60]. The low residual levels of enzyme activity found in these mutants cannot maintain a sufficient concentration of NADPH, meaning that cells cannot even protect themselves against oxygen radicals continuously generated by the normal metabolism in the RBCs. As in other chronic hemolytic anemia, individuals that present a history of neonatal jaundice and chronic anemia are the most clinically severe and may be transfusion-dependent [69]. However, individuals with these Class I mutations represent a minority of the population affected by G6PD deficiency (almost always males). Interestingly, there is a group of 95 Class I mutations that are located mainly in the exon 10, encoding for the dimer interface and structural NADP^+^ of the native G6PD enzyme [34,63,64,65] (Figure 6). The latter region is considered important for the stability and integrity of the functional enzyme [65]. As a consequence, many studies have focused on the protein level to examine the molecular mechanisms regarding the reduced G6PD activity observed in individuals.

## 6. Conclusions

We are updating 16 new mutations that have not been considered in the most recent review and 15 mutations that have been recently described. Most of these variants (11) are considered as Class I, which is the most severe symptomatic group. We localize several of these mutations using bioinformatics tools in the solved three-dimensional structure of the human G6PD protein. The mutations have been found mainly in the coding regions and are buried in the enzyme; some of these are close to active- and NADPH-binding sites affecting the structure and function of this essential enzyme and leading to a diminution in the efficiency of this essential metabolic enzyme, equivalent to the changes to its own structure and function. The G6PD deficiency produces a wide variety of clinical manifestations, with severe hemolytic anemia being one of the most dangerous. This deficiency is caused by a great variety of mutations, with single nucleotide substitutions being the most frequently described followed by multiple mutations, deletions, and intron’s mutations. The characterization of the mutations responsible for G6PD deficiency, give us an integral vision of the disease and open new possibilities for an opportune detection and treatment of this deficiency.

## Figures and Tables

**Table 1:** A list of G6PD mutations not included in the Minucci review plus those most recently described.

Mutation Name	cDNA Nucleotide Substitution	Codon	Amino Acid Substitution	Exon	Class	Origin’s Country	Reference
**Single Missense Mutations**							
Vietnam 1	7G > A	3	Glu → Lys	2	NR	Vietnam	[50]
México DF	193A > G	65	Thr → Ala	4	NR	Mexico	[58]
Bahia	197T > A	66	Phe → Thr	4	II	Brazil	[51]
Vietnam 2	197T > G	66	Phe → Cys	4	NR	Vietnam	[50]
San Luis Potosi	376A > T	126	Asn → Tyr	5	II	Mexico	[37]
Gaza	536G > A	179	Ser → Asn	6	NR	Palestine	[59]
Herlev	592C > A	198	Arg → Ser	6	I/II	Denmark	[53]
Coimbra	593G > A	198	Arg → His	6	II	India	[45]
San Paulo	660C > G	220	Ile → Met	7	IV	Brazil	[47]
Shanghai	691G > C	231	Ala → Pro	7	NR	Chinese	[48]
Tunisia	737T > C	246	Arg → Leu	7	III	Tunisia	[61]
Zacatecas	770G > T	257	Arg → Leu	7	I	Mexico	[37]
Hamburg	827C > T	276	Pro → Leu	8	I	Germany	[39]
Tunis	920A > C	307	Gln → Pro	9	II	Tunisia	[56]
Nefza	968T > C	323	Leu → Pro	9	III	Tunisia	[56]
Karachi	973G > A	325	Asp → Asn	9	NR	Pakistan	[49]
Quilmes	995C > T	332	Ser → Phe	9	I	Argentine	[52]
Unnamed	1088A > T	363	Asn → Ile	10	I	China	[55]
Veracruz	1094G > A	365	Arg → His	10	I	Mexico	[37]
Unnamed	1187C > G	396	Pro → Arg	10	I	Korea	[54]
Merlo	1226C > A	409	Pro → Gln	10	I	Argentine	[52]
Yucatan	1285A > G	429	Lys → Glu	10	I	Mexico	[37]
Tennessee	1465C > G	422	Leu → Val	10	I	USA	[40]
**Multiple Missense Mutations**							
Taif	516–518 del	174	Gly	6	I	Saudi Arabia	[41]
Sierra Leona	311G > A	104	Arg → His	5	III	Sierra Leone	[46]
	376A > G	126	Asn → Asp				
Palermo	769C > A	257	Arg → Met	7	I	Italy	[38]
	770G > T						
Viangchan + Mahidol	871G > A	291	Val → Met	9, 6	II/III	Thailand	[57]
	487G > A	163	Gly → Ser				
Viangchan + Union	871G > A	291	Val → Met	9, 11	II/III	Thailand	[57]
	1360C > T	454	Arg → Cys				
**Non-Coding Region Substitutions**							
Qingzhen	IVS5-1G > A	-	-	-	NR	-	[60]
Unnamed	IVS-VIII 43G > A	-	-	-	III	Tunisia	[61]
Unnamed	IVS-V 655C > T	-	-	-	III	Tunisia	[61]

NR: Class not reported.

## References

[1] Pai G.S., Sprenkle J.A., Do T.T., Mareni C.E., Migeon B.R. (1980). Localization of loci for hypoxanthine phosphoribosyltransferase and glucose-6-phosphate dehydrogenase 202 and biochemical evidence of nonrandom X chromosome expression from studies of a human X-autosome translocation. Proc. Natl. Acad. Sci. USA.

[2] Luzzatto L., Battistuzzi G. (1985). Glucose-6-phosphate dehydrogenase. Adv. Hum. Genet..

[3] Allahverdiyev A.M., Bagirova M., Elcicek S., Koc R.C., Ates S.C., Baydar S.Y., Yaman S., Abamor E.S., Oztel O.N., Canuto R.A. (2012). Glucose-6-Phosphate Dehydrogenase Deficiency and Malaria: A Method to Detect Primaquine-Induced Hemolysis In Vitro. Biochemistry, Genetics and Molecular Biology.

[4] Szabo P., Purrello M., Rocchi M., Archidiacono N., Alhadeff B., Filippi G., Toniolo D., Martini G., Luzzatto L., Siniscalco M. (1984). Cytological mapping of the human glucose-6-phosphate dehydrogenase gene distal to the fragile-X site suggests a high rate of meiotic recombination across this site. Proc. Natl. Acad. Sci. USA.

[5] Patterson M., Schwartz C., Bell M., Hofker M., Trask B., van den Engh G., Davies K.E. (1987). Physical mapping studies on the human X chromosome in the region Xq27-Xqter. Genomics.

[6] Oberle I., Camerino G., Wrogemann K., Arveiler B., Hanauer A., Raimondi E., Mandel J.L. (1987). Multipoint genetic mapping of the Xq26-q28 region in families with fragile X mental retardation and in normal families reveals tight linkage of markers in q26-q27. Hum. Genet..

[7] Motulsky A.G. (1988). Normal and abnormal color-vision genes. Am. J. Hum. Genet..

[8] Filosa S., Calabro V., Lania G., Vulliamy T.J., Brancati C., Tagarelli A., Luzzatto L., Martini G.G. (1993). G6PD haplotypes spanning Xq28 from F8C to red/green color vision. Genomics.

[9] Boyer S.H., Graham J.B. (1965). Linkage between the X chromosome loci for glucose-6-phosphate dehydrogenase electrophoretic variation and hemophilia A. Am. J. Hum. Genet..

[10] Arngrimsson R., Dokal I., Luzzatto L., Connor J. (1993). Dyskeratosis congenita: Three additional families show linkage to a locus in Xq28. J. Med. Genet..

[11] Persico M.G., Viglietto G., Martini G., Toniolo D., Paonessa G., Moscatelli C., Dono R., Vulliamy T., Luzzatto L., D’Urso M. (1986). Isolation of human glucose-6-phosphate dehydrogenase (G6PD) cDNA clones: Primary structure of the protein and unusual 5′ non-coding region. Nucleic Acids Res..

[12] Rattazzi M.C. (1968). Glucose 6-phosphate dehydrogenase from human erythrocytes: Molecular weight determination by gel filtration. Biochem. Biophys. Res. Commun..

[13] Vulliamy T.J., D’Urso M., Battistuzzi G., Estrada M., Foulkes N.S., Martini G., Calabrof V., Poggi V., Giordano R., Town M. (1998). Diverse point mutations in the human glucose-6-phosphate dehydrogenase gene cause enzyme deficiency and mild or severe hemolytic anemia. Proc. Natl. Acad. Sci. USA.

[14] Ruwende C., Hill A. (1998). Glucose-6-phosphate dehydrogenase deficiency and malaria. J. Mol. Med..

[15] Janney S.K., Joist J.J., Fitch C.D. (1986). Excess Release of ferriheme in G6PD-deficient erythrocytes: Possible cause of hemolysis and resistance to Malaria. Blood.

[16] Martini G., Toniolo D., Vulliamy T., Luzzatto L., Dono R., Viglietto G., Paonessa G., D’Urso M., Persico M.G. (1986). Structural analysis of the X-linked gene encoding human glucose 6-phosphate dehydrogenase. EMBO J..

[17] Nkhoma E.T., Poole C., Vannappagari V., Hall S.A., Beutler E. (2009). The global prevalence of glucose-6-phosphate dehydrogenase deficiency: A systematic review and meta-analysis. Blood Cells Mol. Dis..

[18] Frank J.E. (2005). Diagnosis and management of G6PD deficiency. Am. Fam. Phys..

[19] Peters A.L., Van Noorden C.J. (2009). Glucose-6-phosphate dehydrogenase deficiency and malaria: Cytochemical detection of heterozygous G6PD deficiency in women. J. Histochem. Cytochem..

[20] Gómez-Manzo S., Terrón-Hernández J., De la Mora-De la Mora I., González-Valdez A., Marcial-Quino J., García-Torres I., Vanoye-Carlo A., López-Velázquez G., Hernández-Alcantara G., Oria-Hernández J. (2014). The stability of G6PD is affected by mutations with different clinical phenotypes. Int. J. Mol. Sci..

[21] Gómez-Manzo S., Marcial-Quino J., Vanoye-Carlo A., Enríquez-Flores S., De la Mora-De la Mora I., González-Valdez A., García-Torres A., Martínez-Rosas V., Sierra-Palacios E., Lazcano-Pérez F. (2015). Mutations of Glucose-6-Phosphate Dehydrogenase Durham, Santa-Maria and A+ Variants Are Associated with Loss Functional and Structural Stability of the Protein. Int. J. Mol. Sci..

[22] Howes R.E., Piel F.B., Patil A.P., Nyangiri O.A., Gething P.W., Dewi M., Hogg M.M., Battle K.E., Padilla C.D., Baird J.K. (2012). G6PD deficiency prevalence and estimates of affected populations in malaria endemic countries: A geostatistical model-based map. PLoS Med..

[23] Lopez R., Cooperman J.M. (1971). Glucose-6-phosphate dehydrogenase deficiency and hyperbilirubinaemia in the newborn. Am. J. Dis. Child..

[24] Au S.W., Naylor C.E., Gover S., Vandeputte-Rutten L., Scopes D.A., Mason P.J., Luzzatto L., Lam V.M., Adams M.J. (1999). Solution of the structure of tetrameric human glucose 6-phosphate dehydrogenase by molecular replacement. Acta Crystallogr. D Biol. Crystallogr..

[25] Howes R.E., Battle K.E., Satyagraha A.W., Baird J.K., Hay S.I. (2013). G6PD Deficiency: Global Distribution, Genetic Variants and Primaquine Therapy. Adv. Parasitol..

[26] Wrigley N.G., Heather J.V., Bonsignore A., de Flora A. (1972). Human erythrocyte glucose 6-phosphate dehydrogenase: Electron microscope studies on structure and interconversion of tetramers, dimers and monomers. J. Mol. Biol..

[27] Turner N.J. (2000). Applications of transketolases in organic synthesis. Curr. Opin. Biotechnol..

[28] McNicholas S., Potterton E., Wilson K.S., Noble M.E.M. (2011). Presenting your structures: The CCP4mg molecular-graphics software. Acta Crystallogr. Sect. D Biol. Crystallogr..

[29] Manganelli G., Fico A., Martini G., Filosa S. (2010). Discussion on pharmacogenetic interaction in G6PD deficiency and methods to identify potential hemolytic drugs. Cardiovasc. Hematol. Disord. Drug Targets.

[30] Cappellini M.D., Fiorelli G. (2008). Glucose-6-phosphate dehydrogenase deficiency. Lancet.

[31] Luzzatto L., Nannelli C., Notaro R. (2016). Glucose-6-Phosphate Dehydrogenase Deficiency. Hematol. Oncol. Clin. N. Am..

[32] Chen E.Y., Cheng A., Lee A., Kuang W.J., Hillier L., Green P., Schlessinger D., Ciccodicola A., D’Urso M. (1991). Sequence of human glucose-6-phosphate dehydrogenase cloned in plasmids and a yeast artificial chromosome. Genomics.

[33] Greene L.S. (1993). G6PD deficiency as protection against falciparum malaria: An epidemiologic critique of population and experimental studies. Am. J. Phys. Anthropol..

[34] Minucci A., Moradkhani K., Hwang M., Zuppi C., Giardina B., Capoluongo P. (2012). Glucose-6-phosphate dehydrogenase (G6PD) mutations database: Review of the “old” and update of the new mutations. Blood Cells Mol. Dis..

[35] Minucci A., Giardina B., Zuppi C., Capoluongo E. (2009). Glucose-6-phosphate dehydrogenase laboratory assay: How, when, and why?. IUBMB Life.

[36] Kwok C.J., Martin A.C., Au S.W., Lam V.M. (2002). G6PDdb, an integrated database of glucose-6-phosphate dehydrogenase (G6PD) mutations. Hum. Mutat..

[37] Vaca G., Arámbula E., Monsalvo A., Medina C., Nuñez C., Sandoval L., López-Guido B. (2003). Glucose-6-phosphate dehydrogenase (G6PD) mutations in Mexico: Four new G6PD variants. Blood Cells Mol. Dis..

[38] Rigano P., Fabiano C., Pojero F., Niceta M., Pecoraro A., Maggio A., Sammarco P. (2010). Glucose 6-phosphate dehydrogenase Palermo R257M: A novel variant associated with chronic non-spherocytic haemolytic anaemia. Br. J. Haematol..

[39] Kordes U., Richter A., Santer R., Schäfer H., Singer D., Sonntag J., Janka G. (2010). Neonatal cholestasis and glucose-6-P-dehydrogenase deficiency. Pediatr. Blood Cancer.

[40] McDade J., Abramova T., Mortier N., Howard T., Ware R.E. (2008). A novel G6PD mutation leading to chronic hemolytic anemia. Pediatr. Blood Cancer.

[41] Mohamed M.M., El-Humiany A.U.-R. (2006). Molecular Characterization of New Variants of Glucose-6-phosphate Dehydrogenase Deficiency Gene Isolated in Western Province of Saudi Arabia Causing Hemolytic Anemia. Pak. J. Biol. Sci..

[42] Gómez-Manzo S., Marcial-Quino J., Vanoye-Carlo A., Serrano-Posada H., González-Valdez A., Martínez-Rosas V., Hernández-Ochoa B., Sierra-Palacios E., Castillo-Rodríguez R.A., Cuevas-Cruz M. (2016). Functional and biochemical characterization of three recombinant human Glucose-6-Phosphate Dehydrogenase mutants: Zacatecas, Vanua-Lava and Viangchan. Int. J. Mol. Sci..

[43] Kiani F., Schwarzl S., Fischer S., Efferth T. (2007). Three-dimensional modeling of glucose-6-phosphate dehydrogenase-deficient variants from German ancestry. PLoS ONE.

[44] Monteiro W.M., Val F.F.A., Siqueira A.M., Franca G.P., Sampaio V.S., Melo G.C., Almeida A.C.G., Brito M.A.M., Peixoto H.M., Fuller D. (2014). G6PD deficiency in Latin America: Systematic review on prevalence and variants. Mem. Inst. Oswaldo Cruz.

[45] Chalvam R., Kedar P.S., Colah R.B., Ghosh K., Mukherjee M.B. (2008). A novel R198H mutation in the glucose-6-phosphate dehydrogenase gene in the tribal groups of the Nilgiris in Southern India. J. Hum. Genet..

[46] Jalloh A., Jalloh M., Gamanga I., Baion D., Sahr F., Gbakima A., Matsuoka H. (2008). G6PD deficiency assessment in Freetown, Sierra Leone, reveals further insight into the molecular heterogeneity of G6PD A. J. Hum. Genet..

[47] Oliveira R.A., Oshiro M., Hirata M.H., Hirata R.D., Ribeiro G.S., Medeiros T., Barretto O.C. (2009). A novel point mutation in a class IV glucose-6-phosphate dehydrogenase variant (G6PD São Paulo) and polymorphic G6PD variants in Sõ Paulo State, Brazil. Genet. Mol. Biol..

[48] Wang Y.F., Xia W.Q., Ni P.H., Hu Y.Q., Jiang X.C. (2010). Analysis of glucose-6-phosphate dehydrogenase gene mutations: A novel missense mutation. J. Shanghai Jiaotong Univ..

[49] Moiz B., Nasir A., Moatter T., Naqvi Z.A., Khurshid M. (2011). Molecular characterization of glucose-6-phosphate dehydrogenase deficiency in Pakistani population. Int. J. Lab. Hematol..

[50] Hue N.T., Charlieu J.P., Chau T.T., Day N., Farrar J.J., Hien T.T., Dunstan S.J. (2009). Glucose-6-phosphate dehydrogenase (G6PD) mutations and haemoglobinuria syndrome in the Vietnamese population. Malar. J..

[51] Moura Neto J.P., Dourado M.V., Reis M.G., Gonçalves M.S. (2008). A novel c.197T → A variant among Brazilian neonates with glucose-6-phosphate dehydrogenase deficiency. Genet. Mol. Biol..

[52] Chaves A., Eandi S., Defelipe L., Pepe C., Milanesio B., Aguirre F., Feliú-torres A. (2016). Two novel DNA variants associated with glucose-6-phosphate dehydrogenase deficiency found in Argentine pediatric patients. Clin. Biochem..

[53] Warny M., Lausen B., Birgens H., Knabe N., Petersen J. (2015). Severe G6PD Deficiency Due to a New Missense Mutation in an Infant of Northern European Descent. J. Pediatr. Hematol. Oncol..

[54] Jang M.A., Kim J.Y., Lee K.O., Kim S.H., Koo H.H., Kim H.J. (2015). A novel de novo mutation in the G6PD gene in a korean boy with glucose-6-phosphate dehydrogenase deficiency: Case report. Annal. Clin. Lab. Sci..

[55] Chen X., Lv R., Wen F., Chen Y., Liu F. (2016). A Novel A1088T Mutation in the Glucose-6-Phosphate Dehydrogenase Gene Detected by RT-PCR Combined with DNA Sequencing. Ind. J. Hemat. Blood Transf..

[56] Benmansour I., Moradkhani K., Moumni I., Wajcman H., Hafsia R., Ghanem A., Préhu C. (2013). Two new class III G6PD variants (G6PD Tunis (c.920A > C: p.307Gln > Pro) and G6PD Nefza (c.968T > C: p.323 Leu > Pro)) and overview of the spectrum of mutations in Tunisia. Blood Cells Mol. Dis..

[57] Nantakomol R.P., Attakorn P., Day N.P.J., White N.J., Imwong M. (2013). Evaluation of the phenotypic test and genetic analysis in the detection of glucose-6-phosphate dehydrogenase deficiency Duangdao. Malar. J..

[58] García-Magallanes N., Luque-Ortega F., Aguilar-Medina E.M., Ramos-Payán R., Galaviz-Hernández C., Romero-Quintana J.G., Arámbula-Meraz E. (2014). Glucose-6-phosphate dehydrogenase deficiency in Northern Mexico and description of a novel mutation. J. Genet..

[59] Sirdah M., Reading N.S., Vankayalapati H., Perkins S.L., Shubair M.E., Aboud L., Prchal J.T. (2012). Molecular heterogeneity of glucose-6-phosphate dehydrogenase deficiency in Gaza Strip Palestinians. Blood Cells Mol. Dis..

[60] Wei-Liang L., Fang L. (2012). Glucose-6-Phosphate Dehydrogenase Qingzhen: Identification of a Novel Splice Mutation (IVS5–1G > A). Pediatr. Blood Cancer.

[61] Bendaoud B., Hosni I., Mosbahi I., Hafsia R., Prehu C., Abbes S. (2013). Three new mutations account for the prevalence of glucose 6 phosphate deshydrogenase (G6PD) deficiency in Tunisia. Pathol. Biol..

[62] Beutler E., Vulliamy T.J. (2002). Hematologically important mutations: Glucose-6-phosphate dehydrogenase. Blood Cells Mol. Dis..

[63] Mehta A., Mason P.J., Vulliamy T.J. (2000). Glucose-6-phosphate dehydrogenase deficiency. Baillieres Best Pract. Res. Clin. Haematol..

[64] Wang X.T., Chan T.F., Lam V., Engel P. (2008). What is the role of the second “structural” NADP^+^-binding site in human glucose-6-phosphate dehydrogenase?. Protein Sci..

[65] Au S.W.N., Gover S., Lam V., Adams M. (2000). Human glucose-6-phosphate dehydrogenase: The cristal structure reveals a structural NADP^+^ molecule and provides. Structure.

[66] Mason P.J., Bautista J.M., Gilsanz F. (2007). G6PD deficiency: The genotype-phenotype association. Blood Rev..

[67] Filosa S., Calabrò V., Vallone D., Poggi V., Mason P., Pagnini D., Alfinito F., Rotoli B., Martini G., Luzzatto L. (1992). Molecular basis of chronic non-spherocytic haemolytic anaemia: A new G6PD variant (393 Arg–His) with abnormal KmG6P and marked in vivo instability. Br. J. Haematol..

[68] Filosa S., Cai W., Galanello R., Cao A., de Mattia D., Schettini F., Martini G. (1994). A novel single-base mutation in the glucose 6-phosphate dehydrogenase gene is associated with chronic non-spherocytic haemolytic anaemia. Hum. Genet..

[69] Luzzatto L., Mehta A., Vulliamy T., Scriver C.R., Beaudet A.L., Sly W.S., Valle D. (2001). Glucose 6-phosphate dehydrogenase deficiency. The Metabolic and Molecular Bases of Inherited Disease.

[70] Beutler E. (2008). Glucose-6-phosphate dehydrogenase deficiency: A historical perspective. Blood.

[71] Luzzatto L. (2006). Glucose 6-phosphate dehydrogenase deficiency: From genotype to phenotype. Haematologica.

[72] Johnson L.H., Bhutani V.K. (2002). System-based approach to management of neonatal jaundice and prevention of kernicterus. J. Pediatr..

[73] Luzzatto L., Poggi V.E., Orkin S.H., Nathan D.G., Ginsburg D., Look A.T., Fisher D.E., Lux S. (2009). Glucose 6-phosphate dehydrogenase deficiency. Nathan and Oski’s Hematology of Infancy and Childhood.

[74] Maisels M.J. (2006). Neonatal jaundice. Pediatr. Rev..

[75] Carette C., Dubois-Laforgue D., Gautier J.F., Timsit J. (2011). Diabetes mellitus and glucose-6-phosphate dehydrogenase deficiency: From one crisis to another. Diabetes Metab..

[76] Beutler E. (1984). Red Cell Metabolism: A Manual of Biochemical Methods.

